# Readability of Online Patient Education Materials for the 10 Most Common Hand Conditions

**DOI:** 10.7759/cureus.63820

**Published:** 2024-07-04

**Authors:** Cameron Gerhold, Taylor Blount, Michael J Sweeney, Joao Panattoni

**Affiliations:** 1 Orthopedic Surgery, Florida State University College of Medicine, Tallahassee, USA; 2 Plastic Surgery, Florida State University College of Medicine, Tallahassee, USA; 3 Clinical Sciences, Florida State University College of Medicine, Tallahassee, USA; 4 Hand Surgery, Vero Orthopaedics, Vero Beach, USA

**Keywords:** reading level, hand conditions, google, flesch-kincaid, readbility

## Abstract

Background

Millions of individuals every day turn to the internet for assistance in understanding their hand conditions and potential treatments. While online educational resources appear abundant, there are concerns about whether resources meet the readability recommendations agreed upon by the American Medical Association (AMA) and the National Institutes of Health (NIH). Identifying educational resources that are readable for the majority of patients could improve a patient's understanding of their medical condition, subsequently improving their health outcomes.

Methods

The readability of the top five websites for the 10 most common hand conditions was examined using the Flesch-Kincaid (FK) analysis, comprising the FK reading ease and FK grade level. The FK reading ease score is an indicator of how difficult a text is to comprehend, while the FK grade level score is the grade level an individual reading a particular text would need to fully understand the text.

Results

The average FK reading ease was 56.00, which correlates with “fairly difficult (high school)”. The average FK corresponded to an eighth-grade reading level, far above the sixth-grade reading level recommendation set by the AMA and NIH.

Conclusion

Patient education, satisfaction, and the patient-physician relationship can all be improved by providing patients with more readable educational materials. Our study shows there is an opportunity for drastic improvement in the readability of online educational materials. Guiding patients with effective search techniques, advocating for the creation of more readable materials, and having a better understanding of the health literacy barriers patients face will allow hand surgeons to provide more comprehensive care to patients.

## Introduction

With the emergence of the internet, patients increasingly seek information about their diagnosis outside of traditional healthcare settings. It is estimated that nearly six million patients search for health information online every day in the United States alone [1]. The internet serves as a bridge for many patients, filling in the gaps between the information provided by their physician at an appointment and helping to address questions that arise after appointments. Additionally, caregivers may also look online to better understand how they can support the recovery of others, improving their quality of life. While readily available online educational materials empower patients with increased autonomy, not all information is presented in an optimal way for them to understand. In fact, many patients searching for health information online believe the information is of good quality based on whether they can properly evaluate the material [2,3]. This may leave patients feeling defeated, posing a unique challenge for today’s physicians to consider. 

Health literacy is defined as an individual’s ability to find, evaluate, and use information to inform personal health decisions [4]. Approximately 80 million adults in the United States are estimated to have low health literacy levels, with a significant portion of this group consisting of elderly individuals and those with limited formal education [5]. Low health literacy is negatively correlated with a patient’s ability to properly evaluate online health materials [3]. Consequently, it is imperative that online health educational materials are easily understandable for all patients, especially those with lower health literacy. In recent years, there has been a growing emphasis on orthopedic surgeons providing their patients with online educational materials to improve their understanding of their health ailments. When done correctly, online educational material can increase patient autonomy and aid patients in making informed decisions regarding their health. However, the effectiveness of these online materials is entirely dependent on their readability and the literacy level of the intended audience. The readability of a material can be measured using various metrics. The Flesch-Kincaid (FK) readability scores, grade level and reading ease, are the single most used measure of readability and were adopted by the United States military to evaluate the readability of their instructional manuals [6]. The reading level of the average American equates to an eighth-grade reading level [7], underscoring the vital importance of readability in healthcare communications. 

The American Medical Association (AMA) and National Institutes of Health (NIH) unanimously agree all medical educational material should be written at or below a sixth-grade reading level to improve a patient’s health literacy [8-10]. The United States Department of Education estimates nearly 130 million American adults to have less than a sixth-grade reading level, emphasizing the need for the recommendations of the AMA and NIH to be taken seriously [11]. It is also important to note that a patient’s education level does not necessarily equate to their reading level, as most individuals have much lower reading levels than their technical level of education [9]. 

When searching for information about a specific medical condition, patients may come across content from a myriad of academic institutions, private practice physicians, government organizations, medical journals, online support groups, and various other entities. While online information available to patients is essentially endless, a 2023 study analyzing millions of Google search results found that 27.6% of all clicks were for the first Google search results [12]. Furthermore, the top five search results garnered 69.1% of all clicks, and only 0.63% of Google users clicked on a search result located on the second page [12]. This demonstrates the importance of providing reliable and easily understandable health information within the top five search results on Google. 

This study is based on a study conducted in 2017 that assessed the readability of the 10 most common hand conditions listed by the American Society for Surgery of the Hand (ASSH) [9]: carpal tunnel syndrome, basal joint arthritis of the thumb, de Quervain syndrome, Dupuytren’s contracture, ganglion cysts, hand fractures, trigger finger, extensor tendon injuries, flexor tendon injuries, and mallet finger. The 2017 study calculated readability scores for 100 websites and found less than 30% of all websites included were written at or below an eighth-grade reading level. Additionally, this same study concluded that websites that discussed carpal tunnel syndrome had the highest average grade reading level (10.32), while websites that provided information about hand fractures had the lowest average grade reading level (8.14). We aim to provide an update regarding any changes in readability since that time and analyze the factors that have led to these changes. The aim of this study is to evaluate the readability of the top five search results for the 10 most common hand conditions and provide recommendations for orthopedic surgeons when referring patients to online resources. Our goal is to assist orthopedic surgeons in identifying excellent online resources for their patients and analyzing any changes or lack thereof in the current readability of online materials for these 10 hand conditions compared to the study published in 2017. 

## Materials and methods

The 10 hand conditions included in this study were entered into the search engine, Google (Google Inc., Mountainview, CA), in the same way they appear on the ASSH website: “carpal tunnel syndrome,” “arthritis-base of the thumb,” “de Quervain syndrome,” “Dupuytren’s disease,” “extensor tendon injuries,” “flexor tendon injuries,” “ganglion cysts,” “hand fractures,” “mallet finger,” and “trigger finger.”

All content included in this study was accessed by a single investigator on December 1, 2023, utilizing an incognito search with all cookies, location, and user account information disabled. While searching each condition individually, the five most visited websites for each condition were collected. In total, there were 50 patient education entries, which were analyzed for reading ease and grade level by using the FK analysis. The FK analysis is subdivided into two metrics: FK reading ease and FK grade level. 

The FK reading ease measurement formula, 206.835 - 1.015 × (average number of words per sentence) - 84.6 × (average number of syllables per word), and the FK grade level measurement formula, 0.39 × (average number of words per sentence) + 11.8 × (average number of syllables per word) − 15.59, are two widely used and validated tools created to analyze the readability of an educational text.

The FK reading ease measurement is a score of how easy or difficult a text is to comprehend. Higher FK reading ease measurements correspond to an easier ability to comprehend the text being assessed. This measurement can be interpreted using the following guidelines: 0-29: very difficult (postgraduate); 30-49: difficult (college); 50-59: fair (high school); 60-69: standard (eighth to ninth grade); 70-79: fairly easy (seventh grade); 80-89: easy (fifth to sixth grade); 90-100: very easy (fourth to fifth grade). 

The FK grade level measurement is the grade level an individual reading a text must have completed to fully comprehend the text. If a website receives an FK grade level measurement that ends in a decimal of 0.4 or lower, the grade level is rounded down. If a website receives an FK grade level measurement that ends in a decimal of 0.5 or higher, the grade level is rounded up. For example, an FK grade level measurement of 8.8 requires the reader to have a ninth-grade reading level to understand the reading material, while an FK grade level measurement of 7.4 requires the reader to have a seventh-grade reading level to understand the reading material. 

## Results

**Figure 1:**
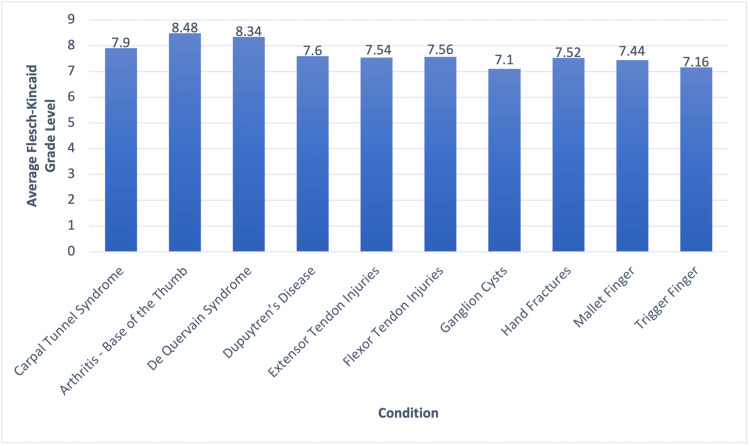
Flesch-Kincaid grade level averages for each condition

**Figure 2:**
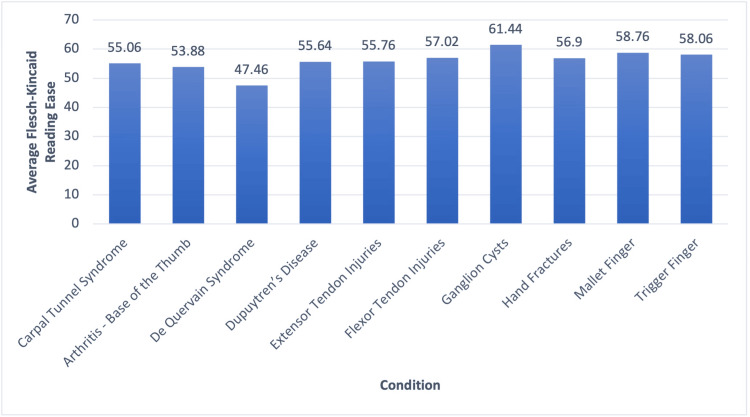
Flesch-Kincaid reading ease averages for each condition

The average FK grade level of the most visited websites for the 10 hand conditions analyzed in this study is displayed in Figure 1. The average FK grade level of all 10 conditions combined was 7.664, a score that requires an eighth-grade reading level. Figure 2 displays the average FK reading ease of the most visited websites for the 10 hand conditions included in this study. The average FK reading ease of all 10 conditions combined was 56.00, which correlates with “fairly difficult (high school)”. Websites on thumb basal joint arthritis had the highest average FK grade level, 8.48, while websites discussing ganglion cysts had the lowest average FK grade level, 7.1. Ganglion cysts also had the highest FK reading ease, with an average of 61.44, corresponding with “standard (eighth to ninth grade)”. De Quervain syndrome had the lowest FK reading ease, with a score of 47.46, corresponding with “difficult (college)”. None of the 50 websites analyzed had FK reading ease scores at or above 70, meaning no website was at or below a seventh-grade level. Additionally, only four out of 50 websites were at or below the recommended sixth-grade reading level (mention this in the introduction as well as who recommends it). Table 1 presents the FK reading ease, FK grade level, organization or practice, and website link for all 50 websites included in this study.

**Table 1:** Flesh-Kincaid (FK) reading ease scores, Flesch-Kincaid grade level scores, and websites analyzed. NIH: National Institutes of Health; AAOS: American Academy of Orthopaedic Surgeons; ASSH: American Society for Surgery of the Hand; BSSH: The British Society for Surgery of the Hand

Organization/Practice	FK reading ease	FK grade level	Google search condition	Website
NIH	50.9	8.8	Carpal tunnel syndrome	https://www.ninds.nih.gov/health-information/disorders/carpal-tunnel-syndrome
Mayo Clinic	51.5	7.4	Carpal tunnel syndrome	https://www.mayoclinic.org/diseases-conditions/carpal-tunnel-syndrome/symptoms-causes/syc-20355603
AAOS	56.3	7.9	Carpal tunnel syndrome	https://orthoinfo.aaos.org/en/diseases--conditions/carpal-tunnel-syndrome/
Johns Hopkins	59.7	6.8	Carpal tunnel syndrome	https://www.hopkinsmedicine.org/health/conditions-and-diseases/carpal-tunnel-syndrome
ASSH	56.9	8.6	Carpal tunnel syndrome	https://www.assh.org/handcare/condition/carpal-tunnel-syndrome
Mayo Clinic	49.5	7.5	Arthritis: base of the thumb	https://www.mayoclinic.org/diseases-conditions/thumb-arthritis/symptoms-causes/syc-20378339
AAOS	57.5	7.4	Arthritis: base of the thumb	https://orthoinfo.aaos.org/en/diseases--conditions/arthritis-of-the-thumb/
ASSH	52.5	8.6	Arthritis: base of the thumb	https://www.assh.org/handcare/condition/thumb-arthritis
Yale	61	9.3	Arthritis: base of the thumb	https://www.yalemedicine.org/conditions/thumb-arthritis
Hospital for Special Surgery	48.9	9.6	Arthritis: base of the thumb	https://www.hss.edu/conditions_basal-joint-arthritis-overview.asp
Mayo Clinic	44.5	8.1	De Quervain syndrome	https://www.mayoclinic.org/diseases-conditions/de-quervain-tenosynovitis/symptoms-causes/syc-20371332
AAOS	53.5	7.8	De Quervain syndrome	https://orthoinfo.aaos.org/en/diseases--conditions/de-quervains-tendinosis
Cleveland Clinic	52.4	8.1	De Quervain syndrome	https://my.clevelandclinic.org/health/diseases/10915-de-quervains-tendinosis
Familydoctor.org	51.4	7.4	De Quervain syndrome	https://familydoctor.org/condition/de-quervains-tenosynovitis/
NIH	35.5	10.3	De Quervain syndrome	https://www.ncbi.nlm.nih.gov/books/NBK442005/
Johns Hopkins	64.2	5.7	Dupuytren’s disease	https://www.hopkinsmedicine.org/health/conditions-and-diseases/dupuytrens-contracture
Mayo Clinic	44.9	8	Dupuytren’s disease	https://www.mayoclinic.org/diseases-conditions/dupuytrens-contracture/symptoms-causes/syc-20371943
AAOS	54	8.4	Dupuytren’s disease	https://orthoinfo.aaos.org/en/diseases--conditions/dupuytrens-disease/
ASSH	62.3	7.5	Dupuytren’s disease	https://www.assh.org/handcare/condition/dupuytrens-contracture
Cleveland Clinic	52.8	8.3	Dupuytren’s disease	https://my.clevelandclinic.org/health/diseases/16941-dupuytrens-contracture
Dr. Gordon Groh	52.6	8.1	Extensor tendon injuries	https://www.drgordongroh.com/orthopaedic-injuries-treatment/hand-wrist/extensor-tendon-injuries
ASSH	66	6.6	Extensor tendon injuries	https://www.assh.org/handcare/condition/extensor-tendon-injury
University of Michigan	61.8	8.2	Extensor tendon injuries	https://www.uofmhealth.org/conditions-treatments/hand-program/extensor-tendon-and-mallet-finger-injuries
BSSH	53	7.1	Extensor tendon injuries	https://www.bssh.ac.uk/patients/conditions/27/extensor_tendon_injury
Orthobullets	45.4	7.7	Extensor tendon injuries	https://www.orthobullets.com/hand/6028/extensor-tendon-injuries
University of Michigan	61.8	8.2	Flexor tendon injuries	https://www.uofmhealth.org/conditions-treatments/hand-program/flexor-tendon-injuries
AAOS	61.4	7.3	Flexor tendon injuries	https://orthoinfo.aaos.org/en/diseases--conditions/flexor-tendon-injuries/
ASSH	57.8	8.2	Flexor tendon injuries	https://www.assh.org/handcare/condition/flexor-tendon-injury
Orthobullets	49.8	7.2	Flexor tendon injuries	https://www.orthobullets.com/hand/6031/flexor-tendon-injuries
BSSH	54.3	6.9	Flexor tendon injuries	https://www.bssh.ac.uk/patients/conditions/26/flexor_tendon_injury
Mayo Clinic	52.5	7	Ganglion cysts	https://www.mayoclinic.org/diseases-conditions/ganglion-cyst/symptoms-causes/syc-20351156
AAOS	60.1	7	Ganglion cysts	https://orthoinfo.aaos.org/en/diseases--conditions/ganglion-cyst-of-the-wrist-and-hand/
Nationwide Children’s Hospital	64.6	5.9	Ganglion cysts	https://www.nationwidechildrens.org/conditions/ganglion-cysts
ASSH	68	6.7	Ganglion cysts	https://www.assh.org/handcare/condition/ganglion-cyst
Penn Medicine	62	8.9	Ganglion cysts	https://www.pennmedicine.org/for-patients-and-visitors/find-a-program-or-service/orthopaedics/hand-and-wrist-pain/treating-ganglion-cysts-in-hand-and-wrist
AAOS	59.2	7.1	Hand fractures	https://orthoinfo.aaos.org/en/diseases--conditions/hand-fractures
Dr. Gordon Groh	47.4	8.7	Hand fractures	https://www.drgordongroh.com/orthopaedic-injuries-treatment/hand-wrist/hand-fractures/
Mayo Clinic	48.5	7.5	Hand fractures	https://www.mayoclinic.org/diseases-conditions/broken-hand/symptoms-causes/syc-20450240
ASSH	67.6	6.1	Hand fractures	https://www.assh.org/handcare/condition/broken-hand
University of Michigan	61.8	8.2	Hand fractures	https://www.uofmhealth.org/conditions-treatments/hand-program/hand-fractures
ASSH	68.5	6.9	Mallet finger	https://www.assh.org/handcare/condition/mallet-finger
AAOS	59.8	7.4	Mallet finger	https://orthoinfo.aaos.org/en/diseases--conditions/mallet-finger-baseball-finger/
Cleveland Clinic	56.7	7.6	Mallet finger	https://my.clevelandclinic.org/health/diseases/21825-mallet-finger
NIH	45.3	9.5	Mallet finger	https://www.ncbi.nlm.nih.gov/books/NBK430811/
Nationwide Children’s Hospital	63.5	5.8	Mallet finger	https://www.nationwidechildrens.org/conditions/mallet-finger
Mayo Clinic	50.8	7.3	Trigger finger	https://www.mayoclinic.org/diseases-conditions/trigger-finger/symptoms-causes/syc-20365100
AAOS	54.6	7.9	Trigger finger	https://orthoinfo.aaos.org/en/diseases--conditions/trigger-finger/
Cleveland Clinic	57.4	7.5	Trigger finger	https://my.clevelandclinic.org/health/diseases/7080-trigger-finger
ASSH	62.7	7.7	Trigger finger	https://www.assh.org/handcare/condition/trigger-finger
WebMD	64.8	5.4	Trigger finger	https://www.webmd.com/rheumatoid-arthritis/trigger-finger

Carpal tunnel syndrome 

The average FK grade reading level of the five most visited websites for carpal tunnel syndrome was 7.9, with a standard deviation of 0.83. All the carpal tunnel syndrome websites were above a sixth-grade reading level, the reading level recommended by the AMA and NIH. However, three of the five websites were at or below the eighth-grade reading level, which is the national average. The average of all five FK reading ease measurements was 55.06, which can be interpreted as “fairly difficult (high school)”. 

Arthritis: base of the thumb 

The average FK grade reading level of the five most visited websites for thumb basal joint arthritis was 8.48, with a standard deviation of 1.01. All websites were above the recommended sixth-grade reading level, and only two websites were at or below an eighth-grade reading level. The mean FK reading ease of the five websites was 53.88, corresponding to “fairly difficult (high school)”. 

De Quervain syndrome 

The average FK grade reading level of the five most visited websites for de Quervain syndrome was 8.34 with a standard deviation of 1.13. All websites were above the recommended sixth-grade reading level, and four websites were at or below an eighth-grade reading level. The mean FK reading ease of the five websites was 47.46, corresponding to “difficult (college)”. 

Dupuytren’s disease 

The average FK grade reading level of the five most visited websites for Dupuytren’s disease was 7.6 with a standard deviation of 1.11. Only one website was at the recommended sixth-grade reading level, but all websites were at or below an eighth-grade reading level. The mean FK reading ease of the five websites was 55.64, corresponding to “fairly difficult (high school)”. 

Extensor tendon injuries 

The average FK grade reading level of the five most visited websites for extensor tendon injuries was 7.54, with a standard deviation of 0.68. None of the five websites were at the recommended sixth-grade reading level, and all websites were at or below an eighth-grade reading level. The mean FK reading ease of the five websites was 55.76, corresponding to “fairly difficult (high school)”. 

Flexor tendon injuries 

The average FK grade reading level of the five most visited websites for flexor tendon injuries was 7.56, with a standard deviation of 0.60. Not one of the five websites analyzed was at the recommended sixth-grade reading level, but all websites were at or below the national reading level average. The mean FK reading ease of the five websites was 57.02, corresponding to “fairly difficult (high school)”. 

Ganglion cysts 

The average FK grade reading level of the five most visited websites for ganglion cysts was 7.1, with a standard deviation of 1.10. Only one website analyzed was at the recommended sixth-grade reading level. All but one website was at or below an eighth-grade reading level. The mean FK reading ease of the five websites was 61.44, corresponding to “standard (8th to 9th grade)”. 

Hand fractures 

The average FK grade reading level of the five most visited websites for hand fractures was 7.52, with a standard deviation of 1.01. One website was at the recommended sixth-grade reading level. All but one website was at or below an eighth-grade reading level. The mean FK reading ease of the five websites was 56.9, corresponding to “fairly difficult (high school)”. 

Mallet finger 

The average FK grade reading level of the five most visited websites for mallet finger was 7.44, with a standard deviation of 1.35. One of the five websites was at the recommended sixth-grade reading level, while four of the five websites were at or below an eighth-grade reading level. The mean FK reading ease of the five websites was 58.76, corresponding to “fairly difficult (high school)”. 

Trigger finger 

The average FK grade reading level of the five most visited websites for trigger finger was 7.16, with a standard deviation of 1.01. One website was at the recommended sixth-grade reading level, while all websites were at or below an eighth-grade reading level. The mean FK reading ease of the five websites was 58.06, corresponding to “fairly difficult (high school)”. 

## Discussion

The plethora of educational websites on hand conditions is a beneficial adjunct to comprehensive care for both patients and orthopedic surgeons. While it can be easy to find useful information about nearly any hand condition, not all top search results offer easily readable material. Our analysis revealed that only five out of the 50 websites assessed had content with reading grade levels at a sixth-grade level or lower. Furthermore, none of the websites analyzed had a reading ease score over 70, indicating that all the websites had a readability level equivalent to 8th grade or higher. 

Websites discussing ganglion cysts, mallet fingers, and trigger fingers had a reading level corresponding to seventh grade, the lowest of all 10 hand conditions in this study. Additionally, websites that provide information on these conditions also scored the highest for reading ease, rating mallet finger and trigger finger as "fairly difficult (high school)," and ganglion cysts as "standard (eighth to ninth grade)." This introduces the question of whether these websites were thoughtfully written for broader patient comprehension or if these hand conditions are simply less complex. 

These results are alarming, as only a few websites met the recommended sixth-grade reading level set by the AMA and NIH for educational materials [8-10]. However, these findings show some improvement compared to a similar study on online educational materials for hand conditions published in 2017, where only five out of 100 websites achieved the targeted sixth-grade reading grade level [9]. Comparing our study with this previous research suggests that there has been some improvement in the readability of materials on hand conditions, although not a significant one. Another important, and possibly promising, discovery was made after examining the conditions with the highest and lowest average grade reading levels within this study and comparing them to the conditions with the highest and lowest average grade reading levels within the 2017 study [9]. The 2017 study found that search results on carpal tunnel syndrome had the highest average grade reading level (10.32), while websites that provided information about hand fractures had the lowest average grade reading level (8.14) [9]. However, the results of this study show websites discussing arthritis at the base of the thumb had the highest average grade reading level (8.48), while websites that provided information about ganglion cysts had the lowest average grade reading level (7.1). Discrepancies in the readability of hand conditions may be due to the overall complexity of the hand condition searched. For example, ganglion cysts are likely a simpler topic for patients to understand in comparison to arthritis at the thumb base. While an exact rationale cannot be given for discrepancies in readability between different hand conditions, these findings may shed light on a trend towards improvement in website readability as the higher average grade reading level in this study (8.48) approaches that of the lowest average grade level in the 2017 study (8.14). A PubMed search of the term “readability” reveals an exponential increase in papers published on this topic, reflecting growing interest within academia. This growth may partly account for the slight enhancement seen in material readability related to common hand conditions over the past seven years. 

Understanding the impact of online educational material readability on millions of patients is an important topic for orthopedic surgeons to appreciate. Providing and recommending readable materials to patients is among the most effective ways for orthopedic surgeons to offer comprehensive care, alleviating patient concerns about various health conditions and strengthening the patient-physician relationship. Surgeons can enhance their ability to effectively explain a condition and its treatment protocol by identifying which materials are accessible to patients. 

Having easily readable online educational materials could also potentially help patients realize their hand conditions are an issue that can be resolved by discussing treatment options with an orthopedic surgeon. Carpal tunnel syndrome is currently one of the most common hand conditions and is the most common entrapment neuropathy, affecting 3%-6% of the general population and having an incidence ranging from 1%-5% [13, 14]. Millions of individuals in the United States alone live with the pain of carpal tunnel syndrome, as it is often an occupational hazard suffered by production workers, office administrative staff, and material movers due to repetitive hand movements [14]. With such a large portion of the population being affected by carpal tunnel syndrome, it is imperative that these individuals are aware their condition can be fixed and they can live a life free from pain caused by carpal tunnel syndrome. To address this issue, orthopedic surgeons should not only recommend legitimate, good-quality websites containing educational materials for their patients’ specific hand conditions but also create educational materials for patients to make information more accessible and readable. 

Patient satisfaction can be improved in many ways, with education being one of the most substantial [15, 16]. The readability of website content can be enhanced by using simplified language, incorporating visual aids like pictures and infographics, and restructuring information with bullet points to optimize understanding and retention of information about hand conditions. 

Orthopedic surgeons and website developers can also consider incorporating multimedia elements such as videos, animations, and interactive graphics to present information in a more engaging and digestible format. These multimedia elements can effectively supplement textual content, providing visual and auditory reinforcement of key concepts and procedures related to hand conditions while simplifying the context through the use of visual aids. Website developers can utilize readability tools, such as the ones used to calculate the readability of websites within this study, to assess the readability of new online patient educational materials prior to publishing these websites. Additionally, the use of multimedia can cater to diverse learning styles, making the educational materials more inclusive and accommodating for a wider range of patients. Lastly, improving the readability of online materials could improve health outcomes and patient satisfaction by allowing patients to play a more collaborative role with their surgeon when discussing the treatment of their medical conditions. 

Finally, orthopedic surgeons and website developers should be mindful of cultural nuances, language preferences, and health literacy levels among different demographic groups. Tailoring the content to resonate with various cultural backgrounds and languages can significantly improve the reach and impact of online educational materials. Additionally, integrating culturally sensitive visual representations and narratives into educational materials can foster greater engagement and resonance among patients from different cultural backgrounds. 

Study limitations

Patients may encounter significant variability in websites when researching common hand conditions, depending on the search engine used. Google search engine was used for this study. While it is the most popular search engine, patients may opt to utilize other search engines, such as Bing or Yahoo, when seeking medical information online. Therefore, we cannot account for any variability that patients may experience when researching the 10 most common hand conditions on a search engine other than Google. Additionally, an individual's Google search engine results are determined by a complex algorithm based on that individual's Google search history, potentially causing differences among the search results of patients with the same hand condition depending on their individual search histories. Another limitation was our scoring metrics, which grade websites on their readability compared to the national average. Many readability tools score websites. We opted to utilize a common readability tool, the FK readability scores, for our study. It is important to note that alternative readability metrics may have provided a more nuanced analysis of the data analyzed in this study. One more limitation was that only the top five websites for each of the 10 most common hand conditions were analyzed. Although this was done purposefully, as the vast majority of individuals who use a search engine only open one of the top five websites that appear, analyzing the readability of more websites that appear for each condition searched may provide readers with a clearer picture of the overall readability of online materials about a condition. Additionally, the use of website design and multimedia elements was not considered in this study. Future studies should be conducted to consider the potential influence of these elements on readability and provide a more comprehensive overview. Lastly, differences in reading level based on socioeconomic status and region are not accounted for in this study, as it is meant to provide a general readability overview.

## Conclusions

In conclusion, the readability of online educational materials for hand conditions plays a crucial role in patient education, satisfaction, and the patient-physician relationship. As evidenced by the findings of this study, there is an opportunity for improvement in ensuring that the information accessible to patients is at an appropriate reading level and presented in a comprehensible manner. Orthopedic surgeons can impact patient care by recommending specific websites with tailored educational materials for their patients' hand conditions. By recommending high-quality websites and advocating for improved website design with enhanced readability features, surgeons can empower patients to access reliable and understandable information about their hand conditions. There should be annual monitoring of the most visited websites providing information about hand conditions to assess whether changes in the readability of online patient educational materials are made. Additionally, there is an opportunity for future studies to explore the readability of patient educational materials that are not accessed online, such as informational pamphlets at a clinic, and determine which elements of educational pamphlets patients respond best to. Continual advancements in the readability of online educational materials for hand conditions will contribute to a more comprehensive approach to orthopedic patient education and care. 

## References

[1] Kanthawala S, Vermeesch A, Given B, Huh J (2016). Answers to health questions: internet search results versus online health community responses. J Med Internet Res.

[2] Morahan-Martin JM (2004). How internet users find, evaluate, and use online health information: a cross-cultural review. Cyberpsychol Behav.

[3] Diviani N, van den Putte B, Giani S, van Weert JC (2015). Low health literacy and evaluation of online health information: a systematic review of the literature. J Med Internet Res.

[4] (2024). What Is health literacy?. https://www.cdc.gov/healthliteracy/learn/index.html.

[5] Hickey KT, Masterson Creber RM, Reading M, Sciacca RR, Riga TC, Frulla AP, Casida JM (2018). Low health literacy: implications for managing cardiac patients in practice. Nurse Pract.

[6] (2024). What is Flesch-Kincaid readability?. https://www.webfx.com/tools/read-able/flesch-kincaid/.

[7] (2024). What is readability and why should content editors care about it?. https://centerforplainlanguage.org/what-is-readability/.

[8] Abu-Heija AA, Shatta M, Ajam M, Abu-Heija U, Imran N, Levine D (2019). Quantitative readability assessment of the internal medicine online patient information on Annals.org. Cureus.

[9] Akinleye SD, Garofolo-Gonzalez G, Montuori M, Culbertson MD, Hashem J, Edelstein DM (2018). Readability of the most commonly accessed online patient education materials pertaining to pathology of the hand. Hand (N Y).

[10] Bluman EM, Foley RP, Chiodo CP (2009). Readability of the patient education section of the AOFAS website. Foot Ankle Int.

[11] (2024). Illiteracy is costing America — here’s why. https://www.usatoday.com/story/sponsor-story/lexia-learning2022/2022/03/02/illiteracy-costing-america-heres-why/6848450001/.

[12] Dean B (2024). We analyzed 5 million Google search results. Here’s what we learned about organic CTR. Backlinko. Published October 14.

[13] Sevy J, Sina R, Varacallo M (2024). Carpal Tunnel Syndrome. https://www.ncbi.nlm.nih.gov/books/NBK448179/.

[14] Joshi A, Patel K, Mohamed A (2022). Carpal tunnel syndrome: pathophysiology and comprehensive guidelines for clinical evaluation and treatment. Cureus.

[15] Chen JG, Zou B, Shuster J (2017). Relationship between patient satisfaction and physician characteristics. J Patient Exp.

[16] Banka G, Edgington S, Kyulo N (2015). Improving patient satisfaction through physician education, feedback, and incentives. J Hosp Med.

